# Rapid detection of cocaine using aptamer-based biosensor on an evanescent wave fibre platform

**DOI:** 10.1098/rsos.180821

**Published:** 2018-10-17

**Authors:** Yong Qiu, Yunfei Tang, Bing Li, Miao He

**Affiliations:** 1 State Key Joint Laboratory of Environment Simulation and Pollution Control, School of Environment, Tsinghua University, Beijing 100084, People's Republic of China; 2 Ecological Environmental Protection Investments Company, China Communications Construction Corporation, Beijing 100013, People's Republic of China; 3 School of Energy and Environmental Engineering, University of Beijing Science and Technology, Beijing 100083, People's Republic of China

**Keywords:** cocaine, competitive affinity, aptamer, evanescent wave fibre biosensor, rapid detection, reproducibility

## Abstract

The rapid detection of cocaine has received considerable attention because of the instantaneous and adverse effects of cocaine overdose on human health. Aptamer-based biosensors for cocaine detection have been well established for research and application. However, reducing the analytic duration without deteriorating the sensitivity still remains as a challenge. Here, we proposed an aptamer-based evanescent wave fibre (EWF) biosensor to rapidly detect cocaine in a wide working range. At first, the aptamers were conjugated to complementary DNA with fluorescence tag and such conjugants were then immobilized on magnetic beads. After cocaine was introduced to compete against the aptamer-DNA conjugants, the released DNA in supernatant was detected on the EWF platform. The dynamic curves of EWF signals could be interpreted by the first-order kinetics and saturation model. The semi-log calibration curve covered a working range of 10–5000 µM of cocaine, and the limit of detection was approximately 10.5 µM. The duration of the full procedure was 990 s (16.5 min), and the detection interval was 390 s (6.5 min). The specified detection of cocaine was confirmed from four typical pharmaceutic agents. The analysis was repeated for 50 cycles without significant loss of sensitivity. Therefore, the aptamer-based EWF biosensor is a feasible solution to rapidly detect cocaine.

## 1. Introduction

*In situ* analysis of cocaine has received considerable attention because of the instantaneous and adverse effects of cocaine overdose on human health [1]. Instrumental analysis, such as liquid chromatography with tandem mass spectrometry (LC-MS/MS) and gas chromatography coupled mass spectrometry (GC-MS), is usually necessary to quantify the trace concentration of cocaine in biological and environmental samples [2,3]. Although the limit of detection (LOD) is satisfactory for cocaine by instrumental analysis, there are challenges on complicated sample pretreatment, expensive instruments and skilled operators are necessary. Thus, portable biosensors have been intensively investigated as they offer attractive advantages over traditional instrumental analysis, such as fast response, easy operation and wide range of analytes [1]. Nowadays, biosensors have been applied to detect various chemical molecules [4], bacteria [5] and pathogens [6].

Aptamers are a class of ligands that can be chemically synthesized to rapidly capture small molecules via target-induced conformational change mechanisms [7], which have been intensively studied and widely used in developing biosensors [8]. The aptamer-target molecular interplay can be transduced to detectable signals, such as fluorescent, colorimetric, electronic and illuminant signals [9–12], favouring real-time and low-cost detection. Other techniques including chemiluminescence [13], enzymatic method [14], evanescent wave [15], quantum dot [16] and impedance [17] have also been successfully applied to detect the transformation of aptamers.

Many efforts have been made to improve the sensitivity of aptameric biosensors. The new structure of aptameric conjugants has attracted researchers' attention to capture cocaine molecules more effectively. Some interesting structures of aptamers, such as rolling circle [18], dual split and triple fragment of aptamers [18–20], can optimize the LOD of cocaine to sub-micromole per litre (micromolar). Another solution involves the enhancement and amplification of the signal transduction by using new materials, e.g. nanoparticles (NPs). Several studies obtained satisfactory cocaine detection performance by using silica, graphene, graphene-gold NPs and fluorescence resonance energy transfer (FRET) to amplify the signals [21–24]. Thus the LOD of cocaine can reach the level of nanomole per litre (nM). Although the sensitivity is greatly improved from classic aptasensors, most of the new protocols remain in conceptual prototypes, which need more verification for practical use.

Optimizing the sensitivity, analytical duration and robustness of aptameric biosensors is still a challenge. Some platforms have been proposed to improve the operation and portability, such as microfluidics [25], optic fibre [15], FRET [26] and portable glucometer [27]. These platforms use *in situ* competition and detection of cocaine, allowing rapid and robust analysis. At the same time, the processes in different kinetics are coupled during the analysis due to the apparatus miniaturization and short duration [9]. For example, 4-parameter logistic model was used in our previous split-aptamer-based all-in-fibre sensor for calibration [20]. In order to decouple the process kinetics, a systematical scheme is requested including high quality of reagents, effective control of environmental conditions and sophisticated models for data interpretation [28]. Moreover, better understanding of the biomolecular competition kinetics is necessary to apply such sensitive and portable system [1].

Here, we proposed an aptamer-based protocol to rapidly detect cocaine in a wide working range on an evanescent wave fibre (EWF) biosensor [29–31], which has been a matured technology to detect small molecules in a wide array of applications [32]. The protocol was validated to achieve acceptable LOD and short duration for cocaine detection. The performance of the EWF biosensor was satisfactory according to selective and reproducible experiments.

## 2. Material and methods

### 2.1. Chemicals

Cocaine was selected as the model pharmaceutic agent. Four pharmaceutics were prepared for selective experiments, including neomycin sulfate, amikacin, sulfadimethoxine and kanamycin sulfate. All pharmaceutics were purchased from a local commercial company (Songyuan Company, Beijing, China). The other chemicals used in experiments, e.g. chemicals in the buffers for analysis, were obtained from a local dealer (Sinapharm Chemical Reagent Company, Shanghai, China). Such chemicals include tris(hydroxymethyl)aminomethane (Tris), ethylene diamine tetra-acetic acid, sodium dodecyl sulfate (SDS), phosphate-buffered saline at pH 7.4 (PBS) and other common reagents.

The aptamer for cocaine is selected from the literature with dissociated coefficient (*K*_d_) as 7 ± 1 µM [33,34]. The oligonucleotides of aptamer, fluorescence-labelled short DNA probe (FSP) and amino-modified anchor DNA probe (AAP) were synthesized and supplied by a local company (Sangon Biotechnology Company, Shanghai, China). All the sequences of the oligonucleotides are shown in table 1. The complementary parts of sequences are bold and underlined. Detailed information on sequence design is available in electronic supplementary material, P1.

**Table 1.** Oligonucleotide sequences used in this study.

name	description	sequence (5′-3′)^a^
aptamer	aptamer of cocaine linked to biotin	biotin-TTTTTTTTTTTTTGA**ATCTCGGGAGAC**AAGGATAAATCCTTCAATGAAGTGGGTCTCCC
FSP	fluorescence-labelled short DNA probe	Cy3-**GTCTCCCGAGAT**
AAP	amino-modified anchor DNA probe	NH_2_-(CH_2_)_6_-TTTTTT**ATCTCGGGAGAC**

^a^The bold and underlined sequence is the complementary part of each probe.

### 2.2. Conjugation of aptamer-FSP

Buffer 1 (hybridization buffer) was used for the solid surface cleaning and aptamer-FSP conjugation, and buffer 2 (reaction buffer) was prepared for the cocaine competition and fibre analysis [19] (electronic supplementary material, M1). The magnetic beads (MB) with diameter of 1 µm were purchased from a supplier abroad (New England Biolabs Inc, MA, USA). The surface of MB was functionalized with biotin-streptavidin for aptamer immobilization. The conjugation of aptamer and FSP on the surface of MB was based on the hybridization method in previous studies [28,35]. Firstly, 500 µl of the MB was washed intensively with buffer 1 five times and stored in a 1.5 ml centrifugal tube after water evacuation. Secondly, 25 µl of the aptamer (4 µM) was mixed with 25 µl of FSP (4 µM). The mixture was warmed at 95°C for 5 min in a water bath and then stored at ambient temperature to assist the conjugation. Thirdly, 50 µl of the aptamer-FSP conjugant was added to the tube with clean MB. Buffer 2 was used to dilute the conjugant into 500 µl of solution, which was further agitated in a thermo-shaker for 30 min to ensure surface stabilization. Finally, the MB was separated from the supernatant, washed five times and stored in 500 µl of buffer 2 for the next step of competition. The activity of MB-aptamer would not be obviously changed after storage at 4°C for at least two weeks [4]. By assuming complete conjugation between the aptamer and FSP, the concentration of FSP was 0.2 µM in the MB solution.

### 2.3. Fabrication of optic fibres

The functionalized optic fibre was fabricated as previously described in the literature [30,31]. Firstly, a multi-mode quartz optic fibre with diameter 600 mm was immersed into piranha solution (30% hydrogen peroxide in concentrated sulfuric acid in v/v = 1 : 3, extreme CAUTION should be used when working with this dangerous mixture) for 1 h to clean and hydroxylate the fibre surface. After that, the optic fibre was treated by 2% (v/v) of 3-aminopropyl triethoxysilane in toluene for 1 h to add amino groups for further reaction. Later, the fibre was introduced to 4% (v/v) glutaraldehyde in pure water to add aldehyde groups to amino groups. Finally, the fibre was immersed into 250 µl of AAP solution (0.25 µM) at 4°C overnight for immobilizing AAP layer on the fibre surface. The fabricated optic fibre can be re-used by simple regeneration using buffer 3 and buffer 4 [20]. Buffer 3 (regeneration buffer) was prepared for the dissociation of FSP-AAP affinity on the fibre surface, and buffer 4 (washing buffer) was used for cleaning afterwards for next cycle of analysis (electronic supplementary material, M1).

### 2.4. Cocaine detection by EWF biosensor

The gradient concentrations of cocaine solutions (0, 10, 25, 50, 100, 250, 500, 1000, 2500 and 5000 µM) were prepared for detection on the EWF biosensor. In each centrifugal tube, 450 µl of cocaine solution was mixed with 50 µl of MB that were immobilized by aptamer-FSP conjugants. After diluting the aptamer-FSP conjugant in MB (0.2 µM) 10 times, the theoretical high limit was 20 nM for FSP to be possibly released to the supernatant. The mixture was agitated in a shaker for 10 min to ensure the complete interaction of cocaine and aptamer [20]. After that, the supernatant was collected in the tube by magnetic separation for 1 min. Part of the supernatant was analysed in a fluorescence spectrometer (F7000, Hitach Corp., Tokyo, Japan) to quantify the released FSP. The supernatant was then injected into the EWF biosensor for analysis with interval of 300 s. After one cycle of detection, the optic fibre was regenerated using buffer 3 and cleansed with buffer 4 for the next cycle, as explained in §2.3. The dynamic signals of fluorescence intensity were recorded and analysed for cocaine quantification. The fluorescence signals at 300 s were used to make the calibration between the cocaine concentrations and the fluorescence intensities.

### 2.5. Data interpretation

To confirm the competition effects between aptamer-MB and cocaine, the supernatant after the competition was directly introduced to a spectrofluorometer to detect the free FSP inside. Assuming that the total active sites of aptamers were limited on MB and constant for cocaine competition, the free FSP would follow the saturation model described as follows:

2.1 $$f=\frac{\;f_{m}\cdot c}{K_{c}+c},$$

where *f* donates the released free FSP, *f*_m_ is the maximum released FSP, *c* is the concentration of cocaine, and *K*_c_ is the half saturation constant. The variables are all expressed in milligram per litre. The parameters *f*_m_ and *K*_c_ can be estimated by using Lineweaver–Burk form of linear fitting.

During cocaine detection, the fluorescence intensity in terms of EWF biosensor voltage signal was governed by the first-order kinetics as previously described [28,30].

2.2 $$I_{t}-I_{0}=I_{m}\cdot (1-e^{-k\cdot t})\;,$$

where *I*_t_ represents the fluorescence intensity at time *t*, *I*_0_ and *I*_m_ are the initial and maximum values of fluorescence intensity, respectively, and *k* is the rate constant (/s). The parameters *I*_m_ and *k* can be determined by the nonlinear fitting of the dynamic curves in Matlab (R2014b, Mathworks, USA). An example was given in electronic supplementary material, figure S2 and Code 1.

### 2.6. Selectivity and reproducibility

The specificity of cocaine detection was verified from four pharmaceutic agents including neomycin, sulfadimethoxine, ampicillin and kanamycin, which are among the frequently used antibiotics [36]. The four agents were prepared in 2000 µM in pure water for the full analytical procedure, whereas 100 µM of cocaine solution was used for comparison. The difference in concentrations made the four agents strong interferences to cocaine detection. The experiments were duplicated to control the data quality. The reproducibility of cocaine detection was examined by repeating the detection of 250 µM cocaine 50 times, which followed the full procedure including competition, detection and fibre regeneration.

## 3. Results and discussion

### 3.1. Scheme of protocol

**Figure 1.**
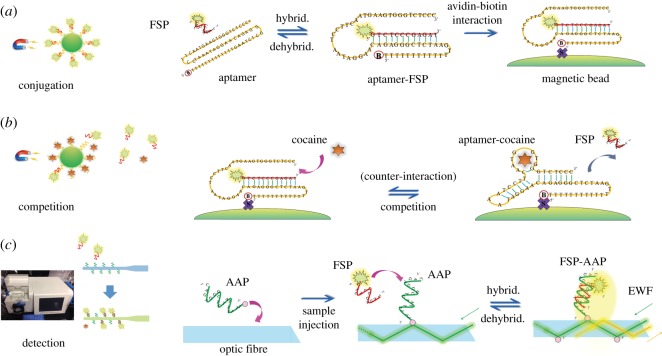
Aptamer-based bioassay of cocaine using an evanescent wave optic-fibre (EWF) biosensor. (*a*) Conjugation step to form MB-aptamer-FSP complex; (*b*) competition step to recognize cocaine and release FSP; (*c*) detection step to quantify the released FSP via fluorescence by evanescent wave excitation on the fibre surface. FSP is fluorescence-labelled probe for quantification, and AAP is complementary probe to anchor FSP on the fibre surface.

The concept of competitive affinity process for cocaine is shown in figure 1. Firstly, the aptamers were conjugated with Cy3-labelled FSP and further immobilized on the MB surface. Secondly, the MB was mixed with cocaine for aptamer competition and FSP was released from the aptamer-FSP conjugants to the bulk solution. Thirdly, the released FSP was introduced to the surface of an optic fibre for the affinity with the AAP. Finally, the amount of FSP-AAP hybrid was quantified by the fluorescence intensity of the evanescent wave. Fluorescence excitation, data recording, and processing were automatically achieved on the integrated EWF biosensor as developed previously [29–31]. Briefly, a laser beam with a wavelength of 535 nm was generated by a pulsed diode and introduced to the optic fibre via total internal reflection, which stimulated evanescent waves at the fibre surface. The fluorophores of FSP-AAP on the fibre surface were excited by the evanescent waves and emitted the fluorescence in 556 nm. The fluorescence was partly transmitted back into the optic fibre and captured by the photodiodes. Together with the EWF biosensor, a fluorescence spectrometer (F7000, Hitachi Company, Japan) was used to provide auxiliary detection.

### 3.2. Competition effect

**Figure 2.**
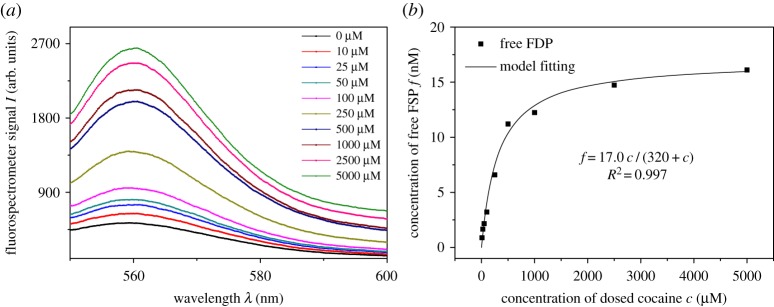
Confirmation of the FSP (fluorescence-labelled short DNA probe) release after competition by cocaine. (*a*) Fluorescent spectra of FSP after competition obtained by fluorospectrometer, *λ* is the wavelength and *I* is the fluorescent intensity; (*b*) the relationship between cocaine concentration and FSP release, *c* is the concentration of cocaine and *f* is the concentration of released FSP.

The strong association of aptamer-FPD conjugant and the easy dissociation of the conjugant by competition should be balanced during analysis. The fluorescence spectra of the supernatant after cocaine competition were obtained by the spectrometer as shown in figure 2*a*. The results showed that the fluorescence intensities of the FSP molecules in the supernatant were positively correlated with the concentration of cocaine in samples, which implied that FSP molecules were successfully released from the aptamer-FSP conjugants on the MB. The FSP concentrations were determined by the calibration curve on the spectrometer (electronic supplementary material, figure S1), and further explained by the saturated model as shown in figure 2*b*. The model explained the data well according to the correlation coefficient (*R*^2^ = 0.997). The maximum FSP (17 nM) in the model implied that the yield of MB-aptamer-FSP conjugation was 85%, considering the theoretical limit of FSP was 20 nM.

### 3.3. Calibration curve and LOD analysis

**Figure 3.**
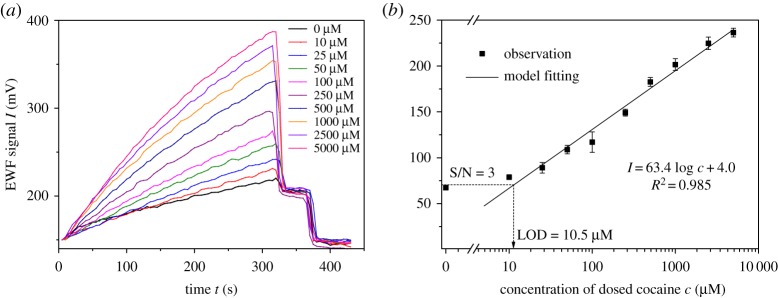
Kinetics of cocaine analysis on the EWF biosensor in full procedure. (*a*) EWF signals along time *t* in gradient cocaine concentrations. (*b*) Calibration curve for cocaine in range of 10–5000 µM fitting by semi-log function. Error bars represent standard deviations of triplicate samples. EWF signals equal to the real-time voltages subtracting the initial voltage. The dashed lines and arrow illustrate the LOD determined by the triplicate blank samples.

The dynamic signals of the EWF biosensor for the detection of cocaine are shown in figure 3*a*. The EWF signal increased with the analytical time after initializing the detection, due to the dynamic affinity between released FSP and immobilized AAP on the fibre surface. After continuous reaction for 300 s, the detection was ceased for fibre surface regeneration using buffers 3 and 4. Thus, the signal dropped to the level lower than control sample by using buffer 3, and reached the baseline by additional cleaning with buffer 4. The analysis was cycled in detection and regeneration for all cocaine samples in sequence.

The signals at the end of detection (300 s) were used to derive the calibration curve as shown in figure 3*b*. The semi-log equation explained the dose–response effects over the full working range from 10 to 5000 µM, with *R*^2^ at 0.985 in the form of normal logarithm. The LOD of cocaine was estimated to be 10.5 µM by three times the standard deviation of the triplicate blank samples, which had an average signal of 67.4 ± 0.5 mV. The increasing signal of the blank sample in figure 3*a* implied possible interferences from the sample matrix, the instrumental noise, transducer bias and the environmental variation. Thus, reducing the signal background is necessary to improve the LOD of EWF biosensor, which should be the further task. Currently, periodic calibration of EWF biosensor is recommended to control the data quality.

### 3.4. Experimental performance of full analytical procedure

One advantage of our protocol is the rapid detection within a very short period (990 s), which consists of 10 min of competition, 5 min of detection and 1.5 min of regeneration. Table 2 lists the durations and LODs of aptamer-based cocaine biosensors in the recent literature. Typical experimental duration was almost 1–2 h for a full procedure. A recent interesting study using FRET enhancement achieved analytical duration within 1000 s [24]; however, an expensive spectrofluorometer with fine temperature control (37°C) was required. Our previous study achieved 450 s for cocaine analysis using split-aptamer on EWF biosensor [20], but skilled operation was required to strictly follow the analytical procedure. The capability of rapid and simple detection with acceptable sensitivity favours our protocol, which allows the use of inexpensive instruments and less human labour.

**Table 2.** Analytical duration of cocaine by aptameric biosensor.

amplification, detection	duration^a^	LODs^b^	reference
aptamer single, fluorescence	—	10 µM	[11]
aptamer single, colorimetry	—	10 µM	[12]
isothermal circular amplification, fluorescence	1 h	0.19 µM	[22]
rolling circle amplification, electrochemistry	>2 h	1.3 nM	[14]
rolling circle amplification, fluorescence	>1 h	0.48 nM	[18]
dual probe of aptamer and hairpin, fluorescence	2 h	2 nM	[37]
silica NP amplification, electrochemiluminescence	1 h	1.3 pM	[38]
aptamer on hairpin, FRET^c^	1000 s	0.2 µM	[24]
split aptamer all-in- fibre, fluorescence	450 s	0.16 µM	[20]
aptamer and optic fibre, fluorescence	990 s	10.5 µM	this study

^a^Sample pretreatment is excluded from the duration.

^b^LOD is the abbreviation of limit of detection and the values are uniformed in micromolar.

^c^FRET is the abbreviation of fluorescence resonance energy transfer.

The LOD of cocaine in this study was approximately 10.5 µM, which is comparable to the reported biosensors of cocaine, e.g. 10 µM by electronics [10] and 10 µM by fluorescence [11] and colorimetry [12]. Usually instrumental analyses with sample extraction own better LOD than biosensors, e.g. 4.9 nM through LC-MS/MS [3] and 19.8 nM via GC-MS [2]. Thus, it is necessary to reduce the LOD of biosensors by various signal amplification techniques, e.g. 0.2 µM by fluorescence quenching [20] and 1.3 nM by enzymatic amplification [14]. LODs lower than 1 nM cocaine are also available by NP enhancement, e.g. 1 nM by graphene-gold NP [21], 0.48 nM by gold NP [18] and 0.29 nM by gold-silica NP [39]. However, expensive materials and advanced instruments are required for the above signal amplification. The previous all-fibre biosensor can detect 0.165 µM of cocaine in serum [20], but the working range is limited below 200 µM and skilled operation is required. Considering that an LOD of 10.5 µM is feasible for the scenarios of rapid screening, the current sensitivity was acceptable by considering its simplicity and rapid operation. Anyway, it is necessary to reduce the background signal in further study, as well as to examine effects of matrices in actual samples by the standard method with recovery rates.

### 3.5. Selectivity and reproducibility

**Figure 4.**
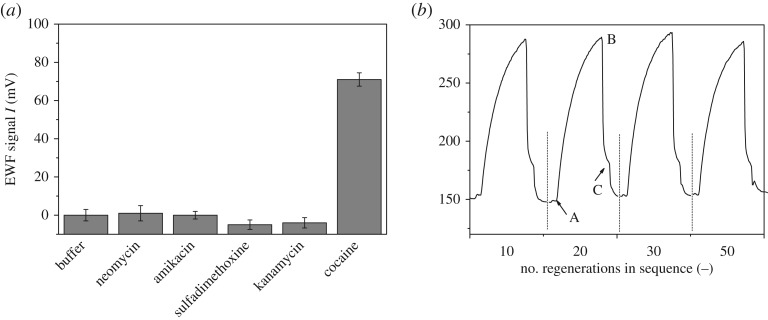
Selectivity and reproducibility of the protocol. (*a*) EWF signal for neomycin, amikacin, sulfadimethoxine and kanamycin at 2000 µM, compared to cocaine at 100 µM. The EWF signal is calculated by subtracting the actual signal of analyte by the control with buffer 2 only. Error bars represent the standard deviations of duplicate samples. (*b*) Repeated analysis of 250 µM cocaine 50 times in full procedure including competition, measuring and fibre regeneration. Only the 10th, 20th, 30th and 50th cycle are shown as examples, with point A: sample injection, point B: regeneration by buffer 3, point C: regeneration by buffer 4.

Selectivity is one of the key indicators in the applicability of developed aptasensor. Figure 4*a* shows the different signals between cocaine and the four other pharmaceutics. A control sample was provided using buffer 2 only. The signal of pharmaceuticals at 300 s on the EWF biosensor was subtracted by that of the control sample. All four pharmaceutics at 2 mM had negligible responses but cocaine sample (0.1 mM) generated an obvious and positive signal (70 mV). The result supported highly specific interaction of aptamers and cocaine molecules [20,27]. Quinine, which was once used to treat malaria and is still used in tonic water, compete the aptamer with strong affinity [40], thus quinine use should be excluded before the detection.

A good regeneration performance is critical for the application of aptasensor. The reproducibility was examined by analysing 250 µM of cocaine 50 times in full procedure. As shown in figure 4*b*, the full dynamic signal curves at the 10th, 20th, 30th and 50th assay cycle were almost the same, indicating good reproducibility of the biosensor with excellent long-term stability of the performance.

## 4. Conclusion

An aptameric biosensor was proposed for the rapid detection of cocaine on the EWF platform. Saturation model and the first-order kinetics were successfully applied to interpret the dynamic EWF signals. The calibration curve in buffer covered a wide working range from 10 to 5000 µM by using the semi-log function for data fitting. The LOD of the method was estimated to be approximately 10.5 µM. The duration of detection was 390 s (6.5 min) and that of the full procedure was 990 s (16.5 min). The reproducibility of the biosensor was confirmed by 50 cycles of analysis without significant loss of performance. The specificity of cocaine by the protocol was verified against four other typical pharmaceutics. The results demonstrated that aptamer-based EWF biosensor has a good potential for the rapid detection of cocaine.

## Supplementary Material

Supplementary Materials

## Data accessibility

Data available from the Dryad Digital Repository: http://dx.doi.org/10.5061/dryad.1j7g2k8 [41].
