# Supplementary material for: Rapid detection of cocaine using aptamer-based biosensor on an evanescent wave fibre platform
Source: R Soc Open Sci. 2018 Oct 17;5(10):180821. doi: 10.1098/rsos.180821 (PMC6227954; doi:10.1098/rsos.180821)
Supplement: Supplementary Materials [file rsos180821supp1.docx]

# Supplementary Materials

# Rapid detection of cocaine using aptamer-based biosensor on an evanescent wave fiber platform

Yong Qiu^1^, Yunfei Tang^1,2*^, Bing Li^3^, Miao He^1*^

^1^ State Key Joint Laboratory of Environment Simulation and Pollution Control, School of Environment, Tsinghua University, Beijing 100084, China

^2^ Ecological Environmental Protection Investments Company, China Communications Construction Corporation, Beijing, 100013, China

^3^ School of Energy and Environmental Engineering, University of Beijing Science and Technology, Beijing, 100083, China

*Corresponding: (Y. Tang) yunfeitang@yeah.net; (M. He) hemiao@tsinghua.edu.cn

Pages: 6

Figures: 2

Methods: 3

Figure S1: Calibration curve of fluorescence-labelled short DNA probe (FSP) by the fluorescence intensity from a fluorospectrometer;

Figure S2: The first order kinetic model fitting of a typical dynamic curve from the evanescent wave fiber (EWF) biosensor;

Method M1: Preparation of buffer solutions;

Method P1: Sequence design of the aptamer and complementary oligonucleotides;

Method Code1: M-file for first-order kinetics fitting in Matlab®.

Figure S1 Calibration curve of fluorescence-labelled short DNA probe (FSP) by the fluorescence intensity from a fluorospectrometer. The signal at 560 nm (*I*) was used to generate the linear calibration, which is *I* = 131·*f*, and *f* is the concentration of FSP. The correlation coefficient R^2^ is 0.999.

Figure S2 The first order kinetic model fitting of a typical dynamic curve from the evanescent wave fiber (EWF) biosensor. The half striation time (t_1/2_) was 32 s, the rate constant *k* was 0.019 s^-1^, and the maximum signal intensity was 804 mV.

**Method M1**: Preparation of buffer solutions

There are four kinds of buffer solutions prepared for the cocaine analysis. Buffer solutions were all prepared from reagent grade chemicals with Millipore ultrapure H_2_O.

Buffer 1 (hybridization buffer) was used for the solid surface cleaning and aptamer conjugation at pH 7.5, containing 0.5 M NaCl, 20 mM Tris in HCl and 1 mM EDTA.

Buffer 2 (reaction buffer) was prepared for the cocaine competition at pH 7.4, which consisted of 25 mM Tris in HCl, 100 mM NaCl, 10 mM KCl and 5 mM MgCl_2_.

Buffer 3 (regeneration buffer) was 0.5% (v/v) SDS in water at pH 1.9 for the fiber surface regeneration.

Buffer 4 (washing buffer) was 10 mM PBS at pH 7.4 for cleaning of the optic fiber surface.

**Method P1:** Sequence design of the aptamer and complementary oligonucleotides

We designed the fluorescence-labeled short DNA probe (FSP) and the amino-modified anchor DNA probe (AAP) to accompany with the structure of the aptamer to recognize cocaine and transduce the signal. The sequences of aptamer, FSP and AAP are shown in Table 1 in the main text.

Here is the explanation on how to design the sequences of the aptamer and complementary oligonucleotides.

1. Aptamer. The aptamer of cocaine has been reported in the literature. Twelve T bases were added in the aptamer sequence to keep the aptamer and FDP away from the surface of magnetic beads (MB) to avoid possible useless combination. Biotin at the end was designed to connect the streptavidin group pre-coated on the surface of MB.

2. FSP. The length of nucleotides (nt) of the FSP probe can greatly affect the aptasensor’s performance, which is usually 8-20 nt. We selected the range of 12 nt at the end of the aptamer’s sequence, thus the FSP probe will be released after the combination of aptamer with cocaine. Fluorophore Cy3 is tailored at the end of FSP.

3. AAP. Six T bases were designed in AAP to make sure the position of fluorophore (Cy3) in the FDP-AAP affinity is close enough to the fiber surface, which is necessary for evanescent wave fluorescence excitation and detection.

Table 1 in the main text

| Name | Description | Sequence (5’-3’) |
| --- | --- | --- |
| Aptamer | Aptamer of cocaine linked to biotin | biotin-TTTTTTTTTTTTTGA**ATCTCGGGAGAC**AAGGATAAATCCTTCAATGAAGTGGGTCTCCC |
| FSP | Fluorescence-labeled short DNA probe | Cy3-**GTCTCCCGAGAT** |
| AAP | Amino-modified anchor DNA probe | NH_2_-(CH_2_)_6_-TTTTTT**ATCTCGGGAGAC** |

**Method Code 1**: M-file for first-order kinetics fitting in Matlab®.

clear;

data=load('data.txt'); %a file of kinetic data was prepared in a matrix.

[row col]=size(data); %read in the data from the text file.

x=data(:,1); %first column in the data.txt is the time.

for i=2:col %other columns in the data.txt are the samples.

y=data(:,i);

fun1=@(b,x) b(1)*(1-exp(-b(2)*x));

%function of *I*_m_·(1− *e*^−k·^*^t^* )

b0=[10 0.01];

% give initial values

[BETA0,R,J,COVB,MSE] = nlinfit(x,y,fun1,b0);

%nonlinear fitting function

outdata(:,i)=[BETA0';MSE];

%record the data including b1, b2 and R2.

end
